# Uniportal thoracoscopic pulmonary lobectomy in the treatment of Lung Cancer

**DOI:** 10.12669/pjms.36.2.793

**Published:** 2020

**Authors:** Jinying Zhang, Haixia Zhao, Lingmei Lv, Jiang Yuan, Yuzhen Sun

**Affiliations:** 1 Jinying Zhang, Department of Cardiothoracic Surgery (B), Binzhou People’s Hospital, Shandong, 256610, China; 2 Haixia Zhao, Department of Breast Surgery, Binzhou People’s Hospital, Shandong, 256610, China; 3 Lingmei Lv, Department of Neurology (B), Binzhou People’s Hospital, Shandong, 256610, China; 4 Jiang Yuan, Department of Cardiothoracic Surgery (B), Binzhou People’s Hospital, Shandong, 256610, China; 5 Yuzhen Sun, Department of Neurosurgery (A), Binzhou People’s Hospital, Shandong, 256610, China

**Keywords:** KEY WORDS: Uniportal thoracoscopy, Triportal thoracoscopy, Lung cancer

## Abstract

**Objective:**

To investigate the clinical efficacy of uniportal thoracoscopic pulmonary lobectomy in the treatment of lung cancer.

**Methods:**

One hundred and ten patients with lung cancer who were admitted to our hospital from February 2017 to June 2018 were enrolled and they were divided into the control group (55 patients) and observation group (55 patients) according to the random number table method. The patients in the observation group received uniportal thoracoscopic pulmonary lobectomy, and patients in the control group underwent triportal thoracoscopic pulmonary lobectomy. The surgical condition, postoperative pulmonary functions, postoperative complication incidence, and postoperative quality of life were compared between the two groups.

**Results:**

The intraoperative blood loss and number of dissected lymph nodes of the observation group were (125.31±12.63) mL and (13.91±2.41) respectively, which were not significantly different with (127.54±13.60) mL and (13.96±2.69) of the control group (P>0.05). The incision length of the observation group was (4.22±0.31) cm, shorter than (6.97±0.42) cm of the control group, the postoperative pain score was (2.87±0.69) points, lower than (4.31±1.09) points of the control group, and the operation time was (195.21±19.42) minutes, longer than (162.68±18.52) min of the control group; the differences were significantly different (P<0.05). The postoperative forced vital capacity (FVC), Maximum Ventilatory Volume (MVV) and Forced Expiratory Volume in 1s (FEV1) in the observation group were (1.90±0.75) L, (54.59±16.03) L/minutes and (1.60±0.53) L respectively, larger than (1.06±0.28) L, (38.41±15.59) L/min and (1.02±0.15) L respectively (P<0.05). The scores of Short Form 36-item Health Survey (SF-36) of patients in the observation group was observed one month after surgery, significantly higher than those in the control group, and the difference was statistically significant (P<0.05). The incidence of complications of the postoperative complication of the observation group was 12.7%, which was not significantly different with 14.5% of the control group (P>0.05).

**Conclusion:**

Patients who receive uniportal video-assisted thoracoscopic pulmonary lobectomy have milder trauma, which is beneficial to the lung functions and postoperative recovery. Moreover, the number of dissected lymph nodes in uniportal thoracoscopic pulmonary lobectomy is equivalent with that in triportal thoracoscopic pulmonary lobectomy. Hence it is worth clinical promotion.

## INTRODUCTION

Lung cancer is a clinically common malignant cancer with high morbidity and mortality.^1^,^2^ The clinical symptoms after onset of lung cancer mainly manifest as hemoptysis, cough, nausea, chest pain, etc. If it is not treated in time, cancer cells will continue to spread and threaten the lives of patients; so early diagnosis and treatment of lung cancer is of great significance to patients. Pulmonary lobectomy is the main surgical method in the treatment of lung cancer.^3^ The traditional open thoracotomy has advantages of wide vision and easy operation, but it may destroy patients’ thoracic integrity and has many postoperative complications and poor prognosis.^4^,^5^ With the rapid development of endoscopic techniques, the emergence of thoracoscopiy has a provided new idea for the clinical treatment of lung cancer. Thoracoscopic surgery includes triportal, biportal and uniportal methods, etc., and previous studies have shown that the efficacy of thoracoscopic surgery is better than that of open thoracoscopic surgery.^6^-^8^ The triportal thoracoscopic pulmonary lobectomy was frequently used in clinics, and in order to further reduce patients’ trauma and pains, the uniportal thoracoscopic pulmonary lobectomy begins to be adopted. A study has pointed out that uniportal thoracoscopic pulmonary lobectomy could reduce the postoperative pains in patients with peripheral lung cancer and shorten the time of postoperative ambulation and hospitalization, and its one-year survival rate reached 85%.^9^

Therefore, in order to further explore the application value and influence of uniportal thoracoscopic pulmonary lobectomy in the treatment of lung cancer, 110 patients with lung cancer who were admitted to our hospital were selected as subjects. The clinical efficacy of uniportal thoracoscopic pulmonary lobectomy and triportal thoracoscopic pulmonary lobectomy was compared.

## METHODS

A total 110 patients with lung cancer who were admitted to our hospital from February 2017 to June 2018 were enrolled and they were divided into control group and observation group according to the random number table method. Patients in the two groups were diagnosed and confirmed as lung cancer and had no severe adhesions in the chest and bronchial invasion. Patients with severe liver and kidney disease, mental illness, other malignant tumors, had less than half a year of expected survival time, or had late-stage lung cancer were excluded. This study was approved by the ethics committee (Ref. No. 126, Dated 2 February 2019) of the hospital, and all the patients in this study have signed relevant informed consent.

All the patients received preoperative cardiopulmonary rehabilitation, smoking cessation and blood pressure and glucose control. Solid food was prohibited six hours before surgery. All patients received general intravenous anesthesia, double-cavity tracheal intubation and one-lung ventilation. Patients took lateral position, with cotton pad under the armpit.

Patients in the control group underwent triportal thoracoscopic pulmonary lobectomy. An incision which was one cm long was made in the 7^th^ or 8^th^ rib along the midaxillary line, and trocar was inserted as a thoracoscopic observation port. A 2 cm incision was made in the 7^th^ or 8^th^ rib along the linea scapularis, and the incision protective sleeve was inserted as the secondary operation port. A 3 ~ 4 cm incision was made in the 4^th^ or 5^th^ rib along the anterior axillary line, and an incision protective sleeve was inserted as the main operation port. Thoracoscopic pulmonary lobectomy and systemic lymphadenectomy were performed, and a chest drainage tube was inserted to the top of the chest through the observation port.

Patients in the observation group had uniportal thoracoscopic pulmonary lobectomy. A 3~4 cm incision was made in the 4^th^ or 5^th^ rib from the midaxillary line to the anterior axillary line, and the incision protective sleeve was inserted as an operation port, through which thoracoscope, aspirator and other instruments were all operated. Thoracoscopic pulmonary lobectomy and systemic lymphadenectomy were performed, and a chest drainage tube was inserted to the top of the chest through the observation port.

### Observation indicators

(1) The surgical conditions including surgery duration, intraoperative blood loss, number of dissected lymph nodes, postoperative pain analog score and length of incision were compared.
(2) The lung function indicators of patients in the two groups, including forced vital capacity (FVC), Maximum Ventilatory Volume (MVV) and Forced Expiratory Volume In 1s (FEV1), were measured by ZJ81Micro Quark pulmonary function device (Cosmed Company, Italy) and compared at the postoperative 3^rd^ day.
(3) The postoperative complications including remant lung infection, remnant lung leakage, pulmonary atelectasis, subcutaneous emphysema, chylothorax, etc. were recorded.
(4) Quality of life was assessed by the 36-item Short Form Health Survey (SF-36) one month after surgery.

### Statistical analysis

The data collected in this study were analyzed by SPSS 22.0. The measurement data was expressed by Mean±SD, and the t test was used for comparison between groups and within group; the enumeration data was expressed as a percentage and processed by Chi-square test. P<0.05 meant that difference was statistically significant.

## RESULTS

There were 55 patients in the control group, including 29 males and 26 females, and they aged from 40 to 72 years old, with an average age of (61.3±1.2) years old. Among them, 11 patients had tumor in the left upper lobe, 14 patients in the left lower lobe, 13 patients in the right upper lobe, 10 patients in the right lower lobe and seven patients in the right middle lobe. The course of disease of them was one to six years, and the average course of disease was (3.12±1.23) years. The diameter of tumor was one to two cm, and the average tumor diameter was (1.5±0.1) cm. There were 55 patients in the observation group, including 28 males and 27 females, and they aged from 41 to 73 years, with an average age of (61.3±1.4) years old. Among them, 12 patients had tumor in the left upper lobe, 13 patients in the left lower lobe, 11 patients in the right upper lobe, 9 patients in the right lower lobe, and 10 patients in the right middle lobe. The course of disease was one to seven years (average (3.13±1.25) years). The tumor diameter was one to two cm (average (1.5±0.1) cm). There were no significant differences in the basic clinical data such as age, tumor diameter, gender, disease course and disease type (P>0.05).

There was no significant difference in the intraoperative blood loss and number of dissected lymph nodes between the two groups (P>0.05). Compared with those in the control group, patients in the observation group had shorter incision length and lower postoperative pain score, but longer operation time, and the differences were statistically significant (P<0.05, Table-I).

**Table-I:** Various surgical indicators of patients between the two groups.

Groups	Observation Group	Control Group	T	P
Duration of surgery (min)	195.21±19.42	162.68±18.52	7.028	<0.05
Length of incision (cm)	4.22±0.31	6.97±0.42	5.813	<0.05
Intraoperative blood loss (mL)	125.31±12.63	127.54±13.60	1.342	>0.05
Postoperative pain score (Point)	2.87±0.69	4.31±1.09	7.426	<0.05
Number of dissected lymph nodes (n)	13.91±2.41	13.96±2.69	0.093	>0.05

The FVC, MVV and FEV1 of the observation group were significantly higher than those of the control group three days after surgery (P<0.05, Table-II).

**Table-II:** Postoperative pulmonary function indicators between the two groups.

Groups	Observation Group	Control Group	t	P
FVC (L)	1.90±0.75	1.06±0.28	10.327	<0.05
MVV (L/min)	54.59±16.03	38.41±15.59	7.411	<0.05
FEV1 (L)	1.60±0.53	1.02±0.15	10.532	<0.05

The postoperative complication incidence was 12.7% in the observation group and 14.5% in the control group. There was no significant difference between the two groups (P>0.05, Table-III).

**Table-III:** Postoperative complication incidence between the two groups.

Groups	Observation Group	Control Group	t	P
Pulmonary atelectasis	1 (1.8)	2 (3.6)	0.479	>0.05
Remnant lung infection	3 (5.5)	3 (5.5)		
Remnant lung leakage	1 (1.8)	1 (1.8)		
Chylothorax	1 (1.8)	1 (1.8)		
Subcutaneous emphysema	1 (1.8)	1 (1.8)		
Complication incidence	7 (12.7)	8 (14.5)		

**Fig.1:**
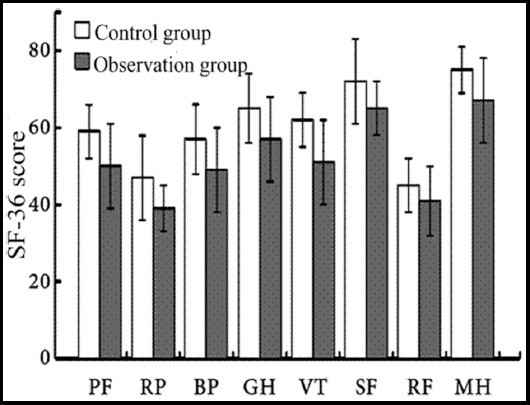
Quality of life scores one month after surgery.

One month after surgery, the self-assessed SF-36 scores of patients, including role physical (RP), body pain (BP), general health status (GH), vitality (VT), etc. in the observation group were significantly higher than those in the control group, and the difference was statistically significant (P<0.05, Fig.1).

## DISSCUSSION

Lung cancer is a type of malignant tumor with a high incidence and mortality.^10^ Smoking is a risk factor for lung cancer and also increases the incidence of lung cancer in passive smokers. The symptoms of lung cancer include local symptoms, systemic symptoms, extrapulmonary symptoms, infiltration and metastasis. For patients with advanced lung cancer, glandular secretion syndrome and neuromuscular syndrome may also appear which has a great impact on patients’ physical and mental health and quality of life.^11^

Thoracoscopic lobectomy is a common surgical method for lung cancer. Traditional multi-portal thoracoscopic lobectomy is mainly triportal, and it is more traumatic, painful, and has more bleeding, which may lead to a series of adverse reactions after surgery; hence patients are difficult to accept.^12^ In 2004, some researchers started to propose uniportal thoracoscopic pulmonary lobectomy, and the efficacy of it was gradually reported in China.^13^,^14^ At present, with the improvement of proficiency of performing U-VATS lobectomy and lymph node dissection and the advancement of the instruments matched with U-VATS, U-VATS indications have gradually expanded.^15^ In 2016, Lyscov et al reported the experience of U-VATS double-sleeve lobectomy and carina reconstruction,^16^ which suggested that U-VATS double-sleeve lobectomy was feasible and effective in the treatment of lung cancer. Compared with the traditional multi-portal thoracoscopic lobectomy, the advantages of uniportal thoracoscopic pulmonary lobectomy are as follows.^17^ Uniportal thoracoscopic pulmonary lobectomy has little effect on patients’ immunity and can prevent the body from severe stress, which will reduce the trauma to the body. Uniportal thoracoscopic pulmonary lobectomy has advantages of small incision, as the surgical injury is mainly concentrated in a certain intercostal space, which effectively reduces the impact of surgery on normal tissues and reduces postoperative pain. The incision of uniportal thoracoscopic pulmonary lobectomy is small, which has little effect on the respiratory functions of patients. It also can promote patients to cough positively, who is beneficial to the recovery of immune functions and reduce the risk of postoperative complications.

A meta-analysis compared the clinical efficacy of U-VATS and M-VATS lobectomy in the treatment of lung cancer.^18^ The results suggested that the postoperative complication incidence, postoperative hospital stay, postoperative chest drainage time and other parameters in the U-VATS group were better than those in the M-VATS, and there was no significant difference in the indicators including surgery-related mortality, surgery duration and blood loss. However, after the accumulation of clinical experience, a recent study proposed that U-VATS had milder trauma and less postoperative pains,^19^ showing an advantage of minimal invasion. The results of this study showed that the observation group had characteristics of longer surgery duration, shorter length of incision and lower postoperative pain scores compared with the control group, and there was no statistical significance in the number of dissected lymph nodes and intraoperative blood loss, which were basically consistent with the conclusions of Wang et al.^20^ It showed that uniportal thoracoscopic pulmonary lobectomy was not significantly different from the triportal thoracoscopic pulmonary lobectomy in the number of dissected lymph nodes with the increase of the proficiency of uniportal thoracoscopic pulmonary lobectomy and the improvement of the instrument but it showed greater advantages in the length of the incision and postoperative pain scores. After lobectomy, the residual volume of the patients increased, and the MVV and FEV1 decreased, which led to hypoxia symptoms, and it was not conducive to the postoperative recovery of body functions.^21^ This study found that the FVC, MVV and FEV1 in the observation group were higher than those in the control group, which suggested that uniportal thoracoscopic pulmonary lobectomy could promote the recovery of lung functions in patients with lung cancer. In addition, this study showed that the postoperative SF-36 scores of the control group were superior than those of the observation group, which suggested that U-VATS pneumonectomy in the treatment of lung cancer was more in line with the concept of minimal invasion and rapid rehabilitation in thoracic surgery and can reduce postoperative pains of patients in the case of skilled operations and improve the quality of life of patients in the near future. Chen et al. found out that the postoperative complication incidence was 9.8% in the observation group and 7.3% in the control group and the difference was not statistically significant,^22^ which was consistent with the results of this study. It indicated that uniportal thoracoscopic pulmonary lobectomy would not increase the incidence of postoperative complications in the treatment of lung cancer.

### Limitations of the study

This study has short follow up time and small sample size; hence the long-term life quality and long-term survival rate of the patients need to be observed by further increasing the sample size to verify its superiority and application values in clinics.

## CONCLUSION

In summary, compared with triportal thoracoscopic pulmonary lobectomy, uniportal thoracoscopic pulmonary lobectomy has the same lymph node dissection effect, but its surgical trauma is mild and the postoperative complication incidence is low, which is beneficial to recovery; hence it has clinical values.

### Authors’ Contribution:

**JYZ & HXZ:** Study design, data collection and analysis.

**HXZ, LML & JY:** Manuscript preparation, drafting and revising.

**JYZ & YZS:** Review and final approval of manuscript, are also responsible for integrity of research.
